# Assessment of a universal preprocedural screening program for coronavirus disease 2019 (COVID-19)

**DOI:** 10.1017/ice.2021.40

**Published:** 2021-02-02

**Authors:** Lana Dbeibo, Kari Kuebler, Alyson Keen, Annie George, Kristen Kelley, Josh Sadowski, Laura Basham, Terrie Beeson, C. Max Schmidt, Cole Beeler, Douglas Webb

**Affiliations:** 1 Indiana University School of Medicine, Indianapolis, Indiana; 2 Indiana University Health Adult Academic Health Center, Indianapolis, Indiana

*To the Editor*—The novel coronavirus 2019 (COVID-19) has caused a global pandemic, placing an unprecedented strain on the US healthcare system. On March 12, 2020, to preserve the safety of hospital staff and patients during the pandemic, the US Department of Health and Human Services and the American College of Surgeons issued a guidance for hospitals and healthcare systems to postpone elective procedures.^2^ Similar guidance followed from the US Surgeon General and the US Centers for Medicare and Medicaid Services, operationalized by individual states.^3,4^ Decreased surgical capacity from COVID-19 has affected healthcare economic and patient outcomes. As a frame of reference, deferred elective surgical activity in 2003 during the severe acute respiratory syndrome (SARS) pandemic resulted in an estimated $32.1 million in direct cost to hospitals in the Toronto and greater Toronto area^5^ and uninteded consequences, such as seriously ill patients not seeking care.^6^

As states have gradually allowed elective procedures to resume in the United States, healthcare organizations have been responsible for mitigating the spread of severe acute respiratory coronavirus virus 2 (SARS-CoV-2), the virus that causes COVID-19. In particular, although the importance of screening all patients with and without symptoms has been recognized, some still question the value of universal screening given economic and operational considerations.

In this study, we aimed (1) to determine the value of universal preprocedural screening for a representative academic health center and (2) to determine the safety of resuming elective procedures using the volume of asymptomatic positive screens.

## Methods

This descriptive study included patients undergoing procedures in the operating room, procedures in the cardiac catheterization lab, and endoscopies at a public, adult, academic, tertiary-care, referral center in Indiana. Patients were included in the sample if they had had a COVID-19 screen performed within 96 hours of a scheduled elective procedure or within 24 hours after an emergent procedure. Patients were classified as symptomatic if they met either of the following criteria: (1) screen performed due to presence of COVID-19 symptoms^7^ or (2) documentation of COVID-19 symptoms in the electronic medical record at the time of the test. Patients with a positive screen that did not meet symptomatic criteria were classified as asymptomatic.

A preprocedural screening program was implemented on May 4, 2020, recommending screening within 96 hours of a scheduled procedure. Screening involved a real-time polymerase chain reaction (RT-PCR) test collected by oropharyngeal and nasopharyngeal swab. Patients with a positive or pending result were rescheduled, unless considered emergent. In the event of an emergent case, COVID-19 isolation precautions were implemented. Standard precautions were followed for patients with a negative screen unless the patient had symptoms and the proceduralist had concern for a false-negative screen.

An infection prevention (IP) data analyst generated a report from the electronic health record for patients undergoing procedures for a 6-week period of time from May 4 through June 14, 2020. An IP and a registered nurse (RN) independently conducted manual chart reviews to verify the inclusion criteria and the screening result and to categorize patients with positive screens as symptomatic or asymptomatic. The IP and RN then cross verified the manual chart reviews to reach consensus, and any discrepancies were resolved by consultation with a third reviewer (an infectious disease physician). Patients meeting symptomatic criteria were excluded from the analysis. Descriptive statistics were used to calculate frequencies and percentages for the included sample of patients.

## Results

The initial sample included 2,194 patients, comprising 46 positive and 2,148 negative screens. Among the 46 positive screens, 29 patients met symptomatic criteria and were excluded from the sample, leaving a final sample of 2,165 patients. The remaining 17 patients were verified as asymptomatic positive screens, resulting in a rate of 0.79% (17 of 2,165). Trauma service patients had the highest positive incidence at 23.5%. Demographic data for the 2,165 patients included in the final sample are provided in Table 1.

**Table 1.** Sample Demographics

Characteristic	Asymptomatic Positive No. (%)	Negative No. (%)	Total No. (%)
Total	17 (0.79)	2,148 (99.21%)	2,165
**Age, y**			
<18		20 (0.93)	20 (0.92)
18–44	11(64.71)	587 (27.33)	598 (27.62)
45–64	5 (29.41)	842 (39.20)	847 (39.12)
65–74	1 (5.88)	438 (20.39)	439 (20.28)
75+		261 (12.15)	261 (12.06)
**Gender**			
Male	9 (52.94)	1,089 (50.70)	1,098 (50.72)
Female	8 (47.06)	1,059 (49.30)	1,067 (49.28)
**Race/Ethnicity**			
Hispanic	2 (11.76)	70 (3.26)	72 (3.33)
Non-Hispanic (NH) White	6 (35.29)	1,664 (77.47)	1,670 (77.14)
NH Black/African American	9 (52.94)	328 (15.27)	337 (15.57)
NH American Indian/Alaska Native		1 (0.05)	1 (0.05)
NH Asian		23 (1.07)	23 (1.06)
NH Native Hawaiian/other Pacific Island		1 (0.05)	1 (0.05)
Not specified		61 (2.84)	61 (2.82)
**Service line**			
Anesthesia	1 (5.88)	4 (0.19)	5 (0.23)
Cardiology	3 (17.56)	18 (0.84)	21 (0.97)
Cardiovascular	1 (5.88)	61 (2.84)	62 (2.86)
Ear, nose, and throat		103 (4.80)	103 (4.76)
Gastroenterology	2 (11.76)	294 (13.69)	296 (13.67)
Neurosurgery	1 (5.88)	125 (5.82)	126 (5.82)
Obstetrics & gynecology	2 (11.76)	148 (6.89)	150 (6.93)
Ophthalmology		2 (0.09)	2 (0.09)
Oral maxillofacial surgery & dentistry		23 (1.07)	23 (1.06)
Orthopedics	2 (11.76)	281 (13.08)	283 (13.07)
Peripheral vascular		74 (3.45)	74 (3.42)
Plastic surgery	2 (11.76)	38 (1.77)	40 (1.85)
Podiatry		5 (0.23)	5 (0.23)
Pulmonology critical care medicine		86 (4.00)	86 (3.97)
Radiology	2 (11.76)	176 (8.19)	178 (8.22)
General surgery	1 (5.88)	340 (15.83)	341 (15.75)
Thoracic		41 (1.91)	41 (1.89)
Transplant		78 (3.63)	78 (3.60)
Urology		251 (11.69)	251 (11.59)

## Discussion

Our study showed a low prevalence of positive asymptomatic COVID-19 screens (0.79%), a rate similar to a preprocedural screening program in the state of Washington (0.8%),^8^ substantially lower than the 5%–80% range reported in an international review.^9^ Notably, however, Indiana was on a downward trend with COVID-19 incidence, decreasing from 15% to 8.1% during the study period.^10^ Despite low incidence of asymptomatic positive cases, our organization continued the preprocedural screening program due to informal feedback indicating proceduralist buy-in, enhanced sense of safety, and improved throughput. Although universal COVID-19 screening might be ideal, this approach may have unintended consequences. For organizations with high surgical volumes, universal screening may increase costs and cause scheduling challenges, and it will likely put additional strain on testing resources for the hospital. Therefore, organizations should consider whether universal screening will produce a high enough yield to offset economic and logistical consequences.

This study had limitations related to generalizability and data analysis. It was conducted at an academic health center in Indiana, and the generalizability of these results to other settings and states with higher incidence may be limited. For example, when this study was conducted, Indiana was on the lower end of case rate per 100,000 (1,611) compared to states with higher rates such as Louisiana (3,431) and Florida (3,114).^1^ In addition, the data analysis focus was descriptive, thus limiting conclusions about relationships and causality or the effects of this program on healthcare worker safety.

Our study validated the value of the preprocedural screening program in allowing the resumption of elective surgical procedures. It was further strengthened through procedural team adoption and sustainment. These findings may help inform decision making of like organizations attempting to enhance safety while resuming elective procedures.
